# The 20 most influential papers produced in the past decade by seven major spinal journals on both altmetric and bibliometric databases

**DOI:** 10.1007/s11845-026-04340-z

**Published:** 2026-04-16

**Authors:** Austin Kerin, Liam O’Dwyer, Peter Dawson, J. Tristan Cassidy

**Affiliations:** 1 University of Galway, Co. Galway, Republic of Ireland; 2 Department of Trauma & Orthopaedic Surgery, University Hospital Limerick, Dooradoyle, Co. Limerick, Republic of Ireland; 3 Royal College of Surgeons, Dublin, Ireland; 4 School of Medicine, University of Limerick, Limerick, Ireland

**Keywords:** Altmetrics, Bibliometrics, Levels of evidence, Spine

## Abstract

**Study design:**

Bibliometric versus altmetric analysis.

**Objectives:**

This study aims to compare the top 20 altmetric articles versus the top 20 bibliometric measures published over the past decade in the world’s top spine journals. The following is hypothesised: 1) The articles in each list will be different; 2) each list will present varying levels of evidence; and 3) each list will reflect distinct geographic and demographic distributions.

**Methods:**

Altmetric Explorer and Web of Science were searched on 4 October 2024 for articles published between 2014–2023 in seven leading spine journals. The top 20 articles by AAS and by citation count were identified. Article type, level of evidence (LOE), citation density, and geographic and demographic engagement were analysed.

**Results:**

No articles overlapped between the two lists. Altmetric articles were mentioned 7,530 times across 107 countries, had a mean citation density of 11.4. Whereas, the bibliometric articles were cited 7,013 times with a mean citation density of 47.5 and only 1010 mentions. Altmetric articles included more level I–II studies (7 vs 1) and were more geographically diverse, with greater representation from developing regions.

**Conclusion:**

Altmetric analysis identified articles with higher LOE and broader dissemination than traditional citation-based metrics. Combining both methods provides a more balanced understanding of research impact in spine surgery.

## Introduction

Research focussing on spine has long been at the forefront of Orthopaedic research and innovation [1] e.g. augmented reality, robotic surgery etc. Historically, bibliometric analysis has been used as a surrogate measure of the influence, value, and quality of academic articles [2]. This approach aggregates citations to calculate an article’s impact factor (IF). The IF often serves as a key metric for researchers when selecting journals for submission, as well as an indicator of attention from the research community [3].

However, the means of information dissemination, both scientific and non-scientific, has changed dramatically over the past 10 years. As a result, alternative means of measuring the influence of scientific works have been developed. The Altmetric Attention Score (AAS), created in 2011, provides a quantitative measure of the online attention a scholarly article receives [4]. This metric captures mentions of scientific outputs on social media platforms, thereby reflecting the impact of newly published articles shortly after their release. Such an approach broadens the evaluation of research impact beyond the confines of traditional bibliometrics.

This study aims to compare the top 20 altmetric articles versus the top 20 bibliometric measures published over the past decade in the world’s top spine journals. The following is hypothesised: 1) The articles in each list will be different; 2) each list will present varying levels of evidence; and 3) each list will reflect distinct geographic and demographic distributions.

## Methods

Two separate searches were conducted on 4th of October 2024. Each search was limited to a time period inclusive of 01/01/2014 and 31/12/2023. A search of the Altmetric database (https://www.altmetric.com/explorer) was by inserting the seven named journals into the “journal or collection section” of the advanced search function on Almetric database: ‘*Spine’, ‘The spine journal’*, *‘Spine deformity’, ‘Journal of spine neurosurgery’*, *‘Clinical spine surgery’, ‘Global spine journal’* and *‘European spine journal’*. The results of this search returned 16,641 items. The title and abstracts were reviewed and from this the top-20 studies were selected for analysis using a bibliometric analysis.

Following on from this the Web of Science database was searched using the following query: Spine (Publication/Source Titles) OR The Spine Journal (Publication/SourceTitles) Spine Deformity (Publication/SourceTitles) OR Journal of Spine Neurosurgery (Publication/SourceTitles) OR Clinical Spine Surgery (Publication/SourceTitles) OR Global Spine Journal (Publication/Source Titles) OR European Spine Journal (Publication/SourceTitles) AND ("2014/01/01"[Date - Publication]: "2023/12/31"[Date - Publication]). This search returned 18,133 results. The results were sorted by citation (highest first). The title and abstracts were reviewed for inclusion and exclusion criteria. The top-20 of these which met the inclusion criteria were selected for analysis and an independent altmetric analysis conducted on these studies.

The following inclusion criteria were applied: 1) studies published in the named peer-reviewed journals within the designated timeframe; 2) Studies in English language. The exclusion criteria was deemed to be anything else not captured by the incluson criteria. All studies involved in spinal research were to be included.

### Analysis of biblometric findings

For the bibliometric search findings the following were recorded: (1) total citations, (2) citation density, (3) authors, (3) title, (4) year of publication, (5) source journal of article, (6) article type (therapeutic study, prognostic study etc.), (7) article subtype (ie, case-series, cohort study, case–control, randomized controlled trial (RCT) etc. (8) geographical location of publication and (9) the level of evidence that was assigned to each clinical research article. The IF for each journal in the results list was also recorded.

### Analysis of almetric findings

For the altmetric search findings the following were recorded: (1) AAS, (2) altmetric source category as outline in Table 1, (3) geographical breakdown, (4) demographic breakdown, (5) mentions per journal.

**Table 1:** Altmetric attention score (AAS) source category weighting

Altmetric source category	AAS weighting
News	8
Blog	5
Policy document (per source); Patent; Wikipedia	3
Peer review (Publons, Pubpeer); Weibo (not trackable since 2019, but historical data kept); Google + (not trackable since 2019, but historical data kept); F1000; Syllabi (Open Syllabus)	1
LinkenIn (not trackable since 2014, but historical data kept);	0.5
X (formerly twitter – posts and re-posts); Facebook (only a curated list of public pages); Reddit; Q&A (Stack Exchan); Youtube	0.25

The level of evidence (LOE) for each article was determined based on the guidelines outlined by the *Journal of Bone and Joint Surgery* [5]. The AAS score is calculated automatically by altmetric.com, considering three key factors: 1) volume (the number of mentions a study receives), 2) sources (the weight assigned to each source, as detailed in Table 1), and 3) authors (the frequency with which the article is referenced by an author, any potential bias, and intended audience of the mentions).

### Data extraction and analysis

**Fig. 1:**
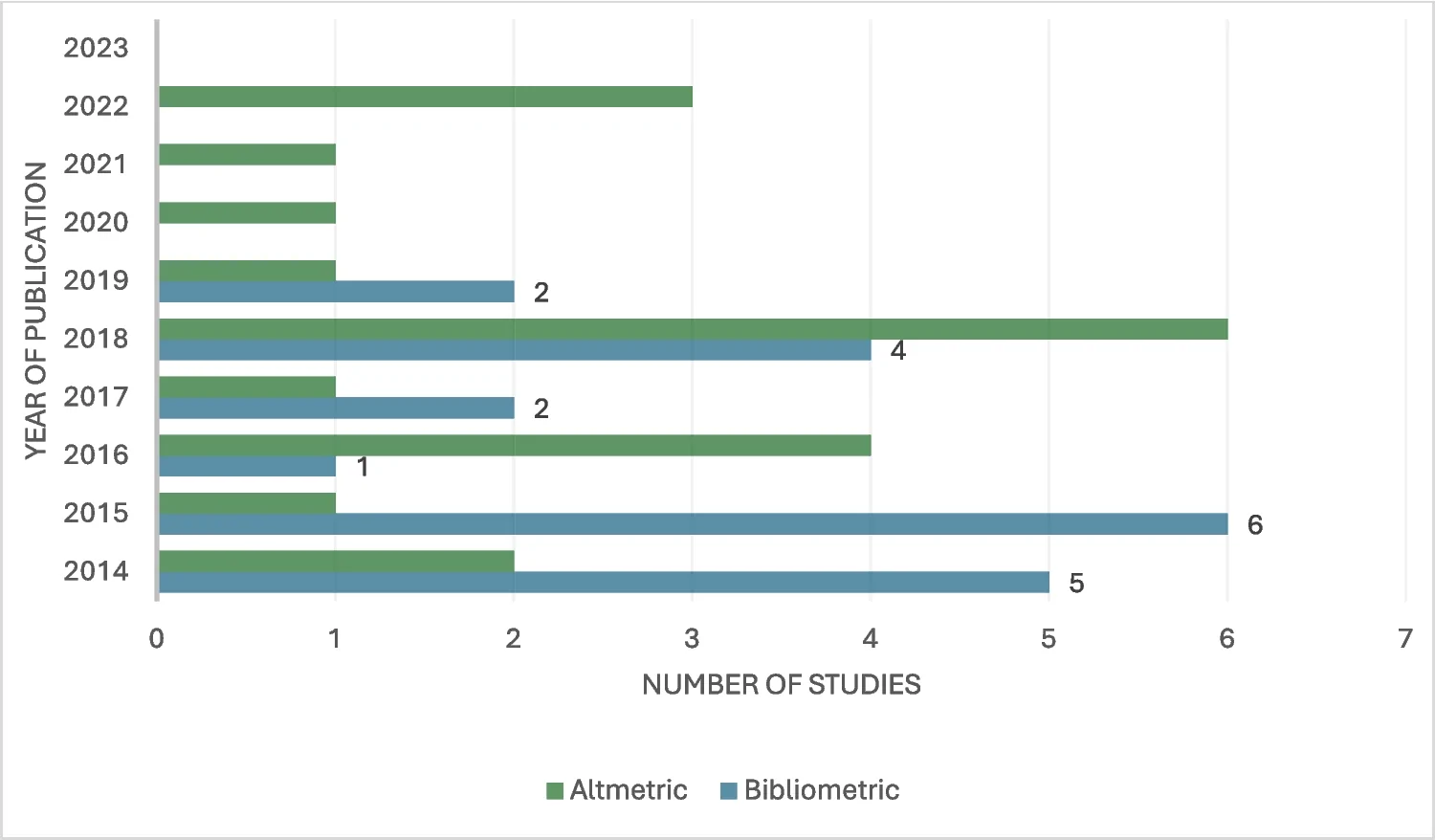
Bar chart showing study publications by year from both altmetric and bibliometric top 20 lists

**Fig. 2:**
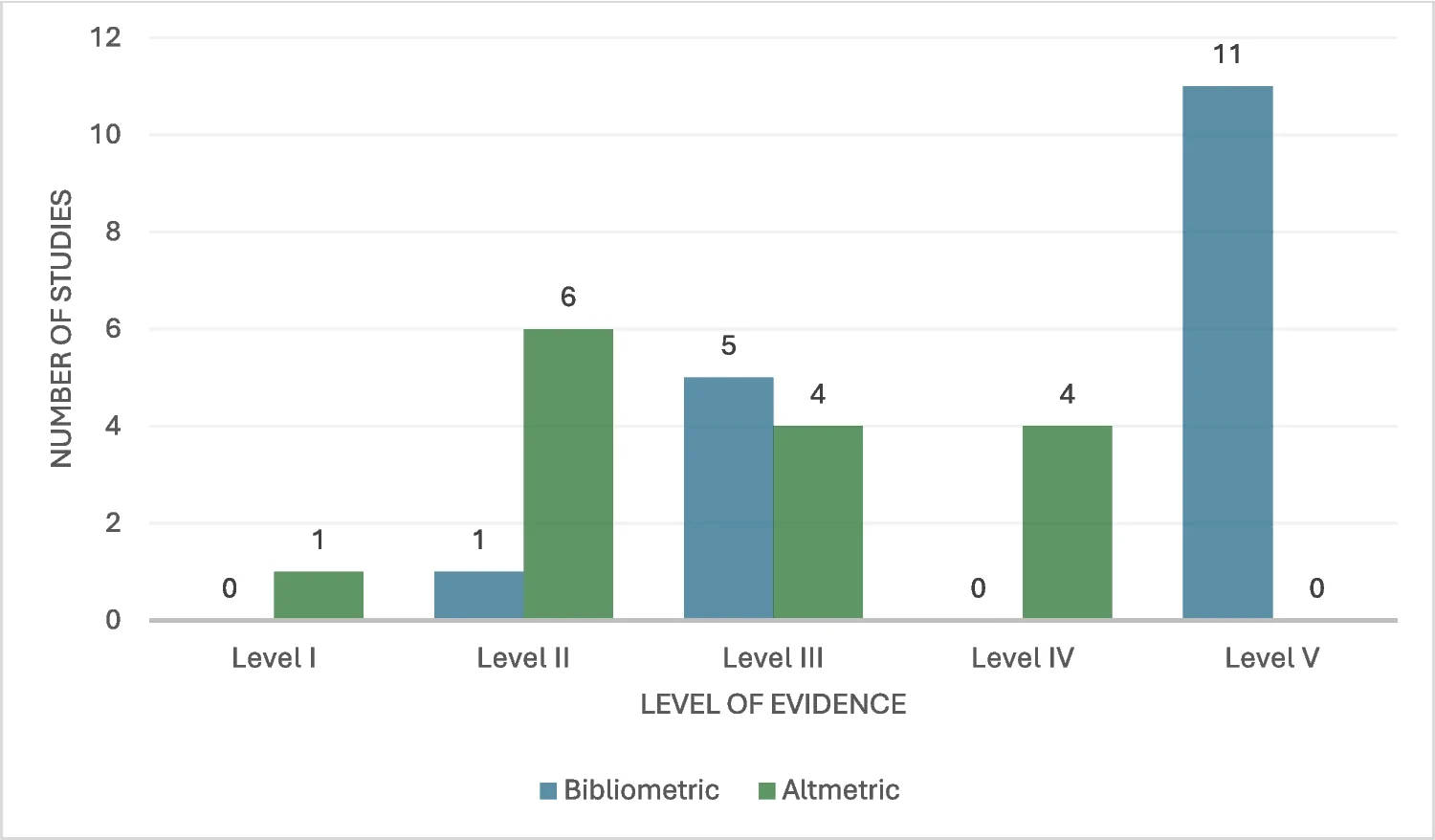
Chart showing the distribution of both altmetric and bibliometric lists and the level of evidence assigned

**Fig. 3:**
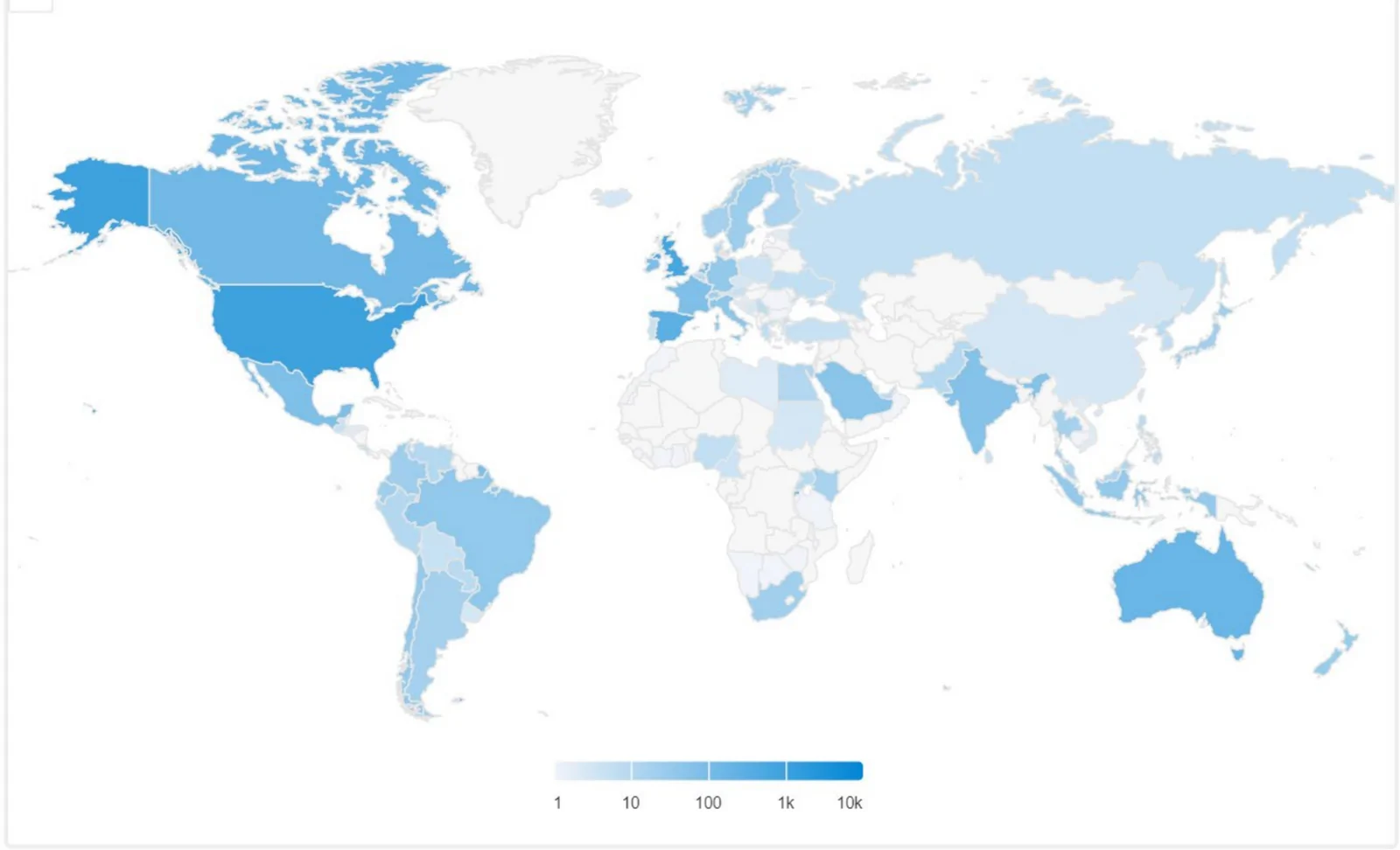
Map of the geographic distribution of X mentions from the top 20 altmetric list

For each article, data were independently extracted by two authors. Discrepancies were resolved by consensus. Citation density was calculated as total citations divided by years since publication. Levels of evidence were assigned using the Journal of Bone and Joint Surgery criteria. Descriptive statistics were performed using means and standard deviations (Figs. 1, 2 and 3).

## Results

### Altmetric explorer search

**Fig. 4:**
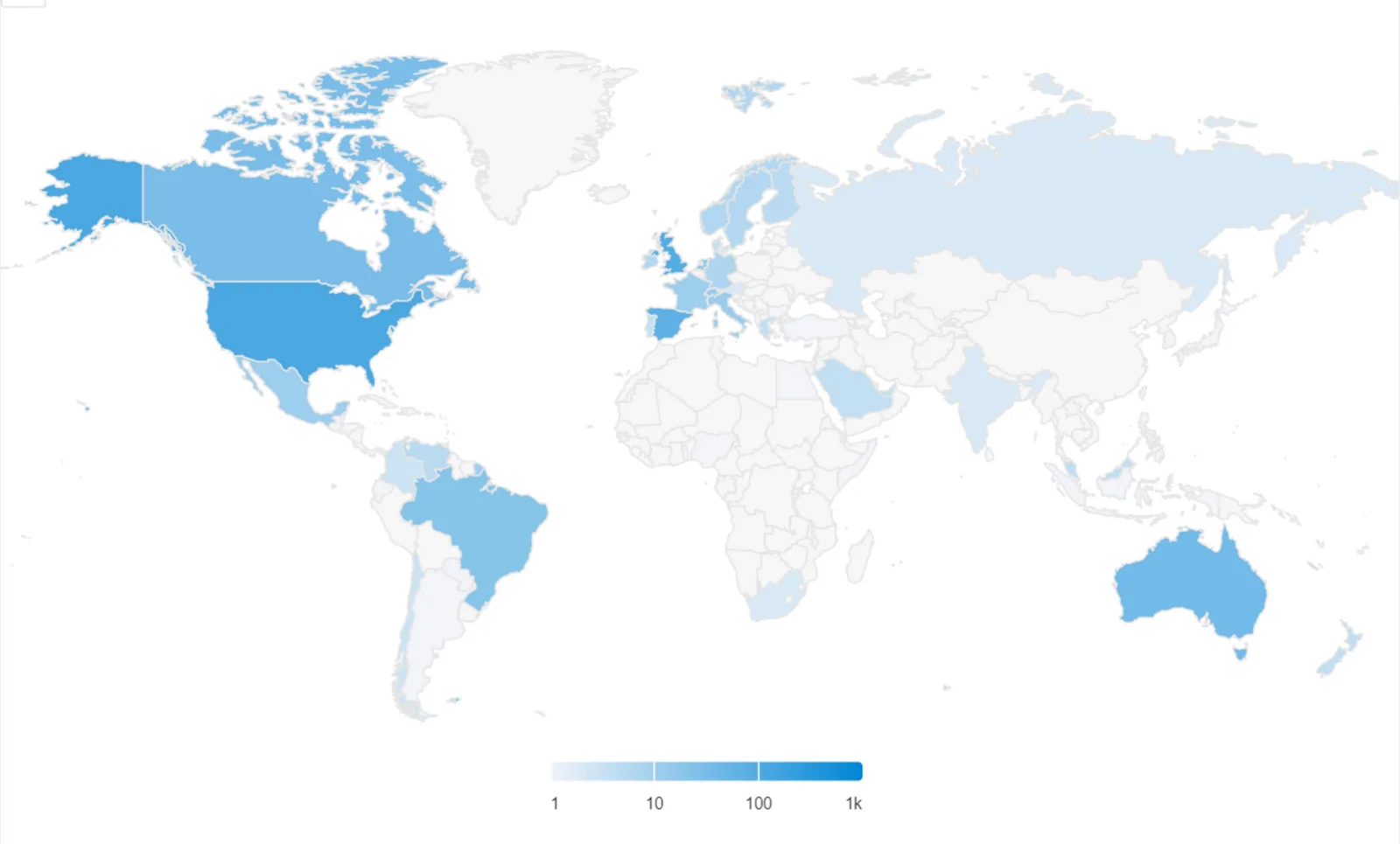
Map of the geographic distribution of X mentions from bibliometric top-20 list

Table 2 outlines the contents of the top 20 altmetric studies. In total the 20 studies were mentioned 7,530 times. The AAS ranged from 1485 to 380, with a mean of 6134 ± 302. These mentions were broken down into 5,776 mentions on the X platform, 1281 news mentions, 475 mentions on the Facebook platform, 27 Google + mentions, 25 blog mentions, and nine Wikipedia mentions. There was discussion of this content in 107 countries by 4,717 unique X users. These countries are represented in Fig. 4. In 2166 (38%) mentions the country could not be determined, from 1,805 (38.3%) unique profiles. Members of the public had by far the most impact on X mentions with 3691 (80%) mentions. This was followed by practitioners with 483 (10.4%) mentions, scientists with 384 (8.3%) mentions and finally science communicators with 58 (1.3%) mentions.

**Table 2:** Top 20 studies according to altmetric attention score (AAS)

Rank	AAS	Title	Citations (DENSITY)
1	1485	Variability in diagnostic error rates of 10 MRI centers performing lumbar spine MRI examinations on the same patient within a 3-week period	41(5)
2	1440	Abnormal Findings on Magnetic Resonance Images of the Cervical Spines in 1211 Asymptomatic Subjects	90(10)
3	932	How did Michael Jackson challenge our understanding of spine biomechanics?	3(0.5)
4	697	Motorcycle helmets and cervical spine injuries: a 5-year experience at a Level 1 trauma center	18(3)
5	637	Treatment of nonsurgical refractory back pain with high-frequency spinal cord stimulation at 10 kHz: 12-month results of a pragmatic, multicenter, randomized controlled trial	34(17)
6	614	Serious adverse event rates and reoperation after arthroscopic shoulder surgery: population based cohort study	105(17.5)
7	564	Does the Level of Cervical Disc Herniation Surgery Affect Performance-based Outcomes in National Football League Athletes?	14(1.75)
8	541	Spinal Cord Injury: The Global Incidence, Prevalence, and Disability From the Global Burden of Disease Study 2019	127(63.5)
9	534	Surgical Navigation Technology Based on Augmented Reality and Integrated 3D Intraoperative Imaging	94(12)
10	510	Overlapping, Masquerading, and Causative Cervical Spine and Shoulder Pathology: A Systematic Review	17(3)
11	496	Negative pressure wound therapy reduces incidence of postoperative wound infection and dehiscence after long-segment thoracolumbar spinal fusion: a single institutional experience	72 (7)
12	481	Exercise as effective as surgery in improving quality of life, disability, and pain for large to massive rotator cuff tears: A systematic review & meta-analysis	31(8)
13	467	The catastrophization effects of an MRI report on the patient and surgeon and the benefits of ‘clinical reporting’: results from an RCT and blinded trials	32(10)
14	451	Augmented Reality Spine Surgery Navigation	37(19)
15	416	Male Spine Motion During Coitus	18(2)
16	415	Lumbar Spine Paraspinal Muscle and Intervertebral Disc Height Changes in Astronauts After Long-Duration Spaceflight on the International Space Station	71(9)
17	412	Cervical disc arthroplasty with the Prestige LP disc versus anterior cervical discectomy and fusion, at 2 levels: results of a prospective, multicenter randomized controlled clinical trial at 24 months	84(12)
18	404	Operative versus nonoperative treatment for the management of full-thickness rotator cuff tears: a systematic review and meta-analysis	53(9)
19	398	A Multicenter Radiographic Evaluation of the Rates of Preoperative and Postoperative Malalignment in Degenerative Spinal Fusions	12(2)
20	380	Prospective, randomized, multicenter study with 2-year follow-up to compare the performance of decompression with and without interlaminar stabilization	35(6)

The most reoccurring article subtype was a retrospective cohort study with six studies. Basic science studies accounted for four of these studies, randomized controlled trials (RCTs) for three and prospective cohort studies and systematic reviews for two each. Case series, cross-sectional study and an editorial piece were all represented by one study each. There were six level II studies, four level III studies, four level IV studies, and one level I studies. Figure 2 outlines the level of evidence attached to each study. It was not possible to assign a level of evidence to every study.

The top-20 studies were cited 988 times, this ranged from 127 to 3. Mean citation count was 49.4 ± 33.9. The citation density ranged from 63.5 to 0.5. The mean citation density was 11.4 ± 63. This is presented in Table 2.

The year of publication ranged from 2014 to 2022. The most represented year was 2018 with six studies, while 2016 followed with four studies and 2022 with three studies.

The most frequently occurring journals on the list was Spine which appeared eight times. This was followed by *Journal of Neurosurgery: Spine* which appeared five times and *Spine Journal* which appeared four times. The *European Spine Journal* and *Global Spine Journal* were represented on two and one occasions respectively.

### Web of science search

Table 3 details the contents of the top 20 bibliometric studies. The AAS ranged from 255 to 1, with a mean of 53.2 ± 65.7. The 1010 mentions received were broken down into 775 (76.7%) mentions on the X platform, 104 news mentions, 73 Facebook mentions, 16 blog mentions, four Wikipedia mentions and four Google + mentions. Of the 775 X posts 447 unique users were involved in 47 different countries. These countries are represented in Fig. 4. There were 176 (36.6%) mentions from 137 (35.7%) profiles which were classified as unknown. Demographically most mentions came from members of the public (432) (67.3%) followed by practitioners themselves (98) (15.2%), scientists (97) (15.1%) and science communicators (14) (2.2%).

**Table 3:** The 20 most-cited articles pertaining to shoulder instability

Rank	Citations (DENSITY)	Title	AAS
1	797 (133)	Clinical practice guidelines for the management of non-specific low back pain in primary care: an updated overview	255
2	588(65)	Degenerative Cervical Myelopathy Epidemiology, Genetics, and Pathogenesis	158
3	528(106)	Trends in Lumbar Fusion Procedure Rates and Associated Hospital Costs for Degenerative Spinal Diseases in the United States, 2004 to 2015	79
4	486(54)	2015 Updated Method Guideline for Systematic Reviews in the Cochrane Back and Neck Group	25
5	377 (38)	An evidence-based clinical guideline for the diagnosis and treatment of lumbar disc herniation with radiculopathy	27
6	348 (58)	National Clinical Guidelines for non-surgical treatment of patients with recent onset low back pain or lumbar radiculopathy	80
7	344 (34)	Lumbar disc nomenclature: version 2.0 Recommendations of the combined task forces of the North American Spine Society, the American Society of Spine Radiology and the American Society of Neuroradiology	170
8	322 (32)	Complications with the use of bone morphogenetic protein 2 (BMP-2) in spine surgery	4
9	295 (30)	Management of thoracolumbar spine fractures	5
10	348(58)	The Global Spine Care Initiative: a summary of the global burden of low back and neck pain studies	104
11	318 (32)	The role of fear avoidance beliefs as a prognostic factor for outcome in patients with nonspecific low back pain: a systematic review	38
12	283 (35)	Defining Spino-Pelvic Alignment Thresholds Should Operative Goals in Adult Spinal Deformity Surgery Account for Age?	1
13	275 (46)	Degenerative Lumbar Spine Disease: Estimating Global Incidence and Worldwide Volume	22
14	252 (28)	Pedicle screw loosening: a clinically relevant complication?	8
15	219(31)	Technical description of oblique lateral interbody fusion at L1-L5 (OLIF25) and at L5-S1 (OLIF51) and evaluation of complication and fusion rates	10
16	264(53)	Sagittal balance of the spine	13
17	261(37)	A Clinical Practice Guideline for the Management of Patients With Degenerative Cervical Myelopathy: Recommendations for Patients With Mild, Moderate, and Severe Disease and Nonmyelopathic Patients With Evidence of Cord Compression	22
18	259(29)	Core outcome domains for clinical trials in non-specific low back pain	21
19	232(26)	Incidence of traumatic spinal cord injury worldwide: a systematic review	11
20	217(24)	Effect of Indirect Neural Decompression Through Oblique Lateral Interbody Fusion for Degenerative Lumbar Disease	11

Of the article subtypes the most prevalent was a clinical guideline being represented five times. This was followed by four literature reviews, two systematic reviews and two retrospective cohort studies. An analysis, prospective clinical study, narrative review and meta -analysis were all represented on one occasion.

Three basic science studies were found on the list. There were 11 level V studies, five level III studies and one level II study on the bibliometric list. Figure 2 outlines the level of evidence attached to each of the studies. Again not at all studies could be assigned a level of evidence.

The top-20 studies were cited 7013 times, ranging from 797 to 217. The mean citation count was 350.7 ± 138.8. The citation density ranged from 133 to 24. The mean citation density was 47.5 ± 109. This is presented in Table 3.

The year of publication ranged from 2014 to 2019. The most represented year was 2015 with six studies followed by 2014 with five studies. There was no representation on the bibliometric list between the years 2020–2023 inclusive.

The most common publishing journal was the *European Spine Journal* with seven studies. *The Spine Journal* was represented by six studies, while *Spine* was represented by 5 studies. Finally, *Global Spine Journal* accounted for two studies.

The IF for each journal were *Spine* 3.5 *The Spine Journal* 4.9, *Spine Deformity* 1.6, *Journal of Neurosurgery: Spine* 2.9, *Clinical Spine Surgery* 1.7, *Global Spine Journal* 3 and *European Spine Journal* 2.6. Three journals were non represented in either list.

## Discussion

The main findings of this study are the altmetric list included studies which were of a higher level of evidence. Furthermore, there was no overlap of studies between the two lists and the altmetric search covered papers across a greater span of the decade. This is in line with previous findings on spinal trauma that there was no correlation between AAS and citations for articles pertaining to spine trauma [6].

The *Spine Journal* had the highest IF (4.9) and appeared four times on the altmetric list and six times on the bibliometric list. However, below this the *Journal of Neurosurgery: Spine* (IF 2.9) failed to show on the bibliometric list and was represented five times on the altmetric list. Interestingly, the most cited study from the bibliometric list produced the highest AAS and came from a journal which did not have the highest IF. This would be in line with the findings from previous studies which found a positive correlation between citations and AAS [7, 8]. However, the study with the highest AAS did not have the highest citations and no study appear on both lists. This means studies with a high citation count may have a high AAS score compared to other highly cited studies, but not when comparing the studies with the highest AAS. Having an article widely mentioned online does not infer greater citations and vice versa, with a greater amount of citations does not mean a greater online presence. These findings are similar to studies looking at shoulder instability and rotator cuff injury [9, 10].

The altmetric list produced more studies of a higher level of evidence. The altmetric list included one level I study and six level II studies, while the bibliometric list had no level I studies and only one level II study. Such results, seem to predict that altmetric scores can be associated with a higher LOE. This is in keeping with similar findings in studies relating to shoulder instability [9, 10]. However, this differs from some publications which found bibliometric and altmetric analysis unrelated to LOE [11]. This distribution of study type reflects the importance of observational studies in spinal research, with retrospective cohort studies offering valuable insights based on real-world data [12]. While RCTs remain the gold standard in clinical research, the low numbers of such studies on both lists seems to suggest a lack of representation of such works on online platforms and declining citation numbers. The regular appearance of cohort studies and systematic reviews may indicate an appetite for evidence generated from non-randomized designs. This is especially important in fields like spine surgery, where long-term outcomes are critical irrespective of level of evidence. However, In order to standardise clinical practice and optimise patient care more level I studies must be produced which will help develop gold standard guidelines for clinical practice.

From a timeframe standpoint there was a much wider range of years covered in the altmetric list. The altmetric system is not time dependant unlike the traditional bibliometric system. As a result it is almost impossible to allow for recently published research to accrue citations to appear on such a list [7]. The ability of members of the public, clinicians, political figures and the scientific community to interact with research online so rapidly enhances the dissemination of information. The ever-expanding presence of social media over the last decade is something researchers must account for. Social media platforms will be influential in policy change, public perception of new innovations/practices, and ultimately funding distribution [3, 13]. Across both datasets members of the public were responsible for most interaction online with research in comparison to medical professionals and others. This demonstrates perhaps a genuine interest in spine research in the public eye which could guide future research and funding in the field [14].

Geographically, the USA, UK and Spain were the top contributors to both lists. The altmetric list, as can be seen from Fig. 4, the developing world was represented relatively well. However, there was poor representation from these countries in the bibliometric list (Fig. 5). Poor representation on academic journals from authors from the developing world has long been noted [15]. A possible explanation for this may be that most of these countries face lower funding levels for research access and fewer opportunities for publication. Additionally, access to orthopaedic care in the developing world is constrained by a lack of facilities, equipment, and properly trained personnel [16, 17]. Given that these areas are where the musculoskeletal burden is greatest, authorship from these areas could potentially be highly beneficial to guide future orthopaedic management [8, 18]. Altmetrics allows us to gauge the research that is currently influencing practice worldwide and not just in a select few countries who have the ability to cite and publish research.

In essence, both parameters set out to achieve different outcomes but both can be brought together to gain a wider appreciation of an article. While citation quantity and AAS may be separate entities, the non-time related bias of AAS make it an important ally to citations when critically analysing a paper in the orthopaedic field [19]. As such, one can see how relevant and accessible research is distributed to an ever more informed public and help guide further research to aid in both patient and physician learning and improve patient outcomes [8]. Altmetrics may serve as an early signal for high-quality, widely disseminated research, while bibliometrics remain valuable for long-term academic influence. Incorporating both may better inform clinicians, researchers, and policymakers when evaluating emerging evidence in spine care.

## Limitations

Both the Altmetric Explorer and the WOS database have limitations in terms of the number of studies they cover, so the articles listed in this study do not represent a comprehensive list. The WOS was selected as the primary database for identifying the most cited articles on spinal pathology, as citation counts varied across different databases. Consequently, other databases like Scopus were excluded. Additionally, only seven key journals focused on spinal literature were included, meaning the results are not exhaustive. The study does not provide information on how the quality of sources is weighted when calculating the AAS. Only English-language studies were included, which may not fully capture the global scope of research on spinal pathology.

## Conclusion

Altmetric analysis identified articles with higher LOE and broader dissemination than traditional citation-based metrics. Combining both methods provides a more balanced understanding of research impact in spine surgery.

## Data Availability

Not applicable in this instance.
